# Assessing Motor Fluctuations in Parkinson’s Disease Patients Based on a Single Inertial Sensor

**DOI:** 10.3390/s16122132

**Published:** 2016-12-15

**Authors:** Carlos Pérez-López, Albert Samà, Daniel Rodríguez-Martín, Andreu Català, Joan Cabestany, Juan Manuel Moreno-Arostegui, Eva de Mingo, Alejandro Rodríguez-Molinero

**Affiliations:** 1 Technical Research Centre for Dependency Care and Autonomous Living, CETPD, Universitat Politècnica de Catalunya, Barcelona Tech., Rambla de l’Exposició 59-69, Vilanova i la Geltrú 08800, Barcelona, Spain; albert.sama@upc.edu (A.S.); daniel.rodriguez-martin@upc.edu (D.R.-M.); andreu.catala@upc.edu (A.C.); joan.cabestany@upc.edu (J.C.); moreno@aha-dee.upc.edu (J.M.M.-A.); 2 Clinical Research Unit, Consorci Sanitari del Garraf (Fundación Sant Antoni Abat ), Carrer de Sant Josep, 21-23, Vilanova i la Geltrú 08800, Barcelona, Spain; itzahe1@hotmail.com (E.d.M.); arodriguez@csg.cat (A.R.-M.)

**Keywords:** inertial sensors, Support Vector Machine, Parkinson’s disease, motor fluctuations, ambulatory monitoring

## Abstract

Altered movement control is typically the first noticeable symptom manifested by Parkinson’s disease (PD) patients. Once under treatment, the effect of the medication is very patent and patients often recover correct movement control over several hours. Nonetheless, as the disease advances, patients present motor complications. Obtaining precise information on the long-term evolution of these motor complications and their short-term fluctuations is crucial to provide optimal therapy to PD patients and to properly measure the outcome of clinical trials. This paper presents an algorithm based on the accelerometer signals provided by a waist sensor that has been validated in the automatic assessment of patient’s motor fluctuations (ON and OFF motor states) during their activities of daily living. A total of 15 patients have participated in the experiments in ambulatory conditions during 1 to 3 days. The state recognised by the algorithm and the motor state annotated by patients in standard diaries are contrasted. Results show that the average specificity and sensitivity are higher than 90%, while their values are higher than 80% of all patients, thereby showing that PD motor status is able to be monitored through a single sensor during daily life of patients in a precise and objective way.

## 1. Introduction

Parkinson’s disease (PD) is a neurodegenerative disorder whose pathology is typified by a deficit of dopamine-producing neurons, which is a neurotransmitter involved in movement control [1]. Probably, the best known and most recognisable symptom of PD are resting tremors. Parkinsonian tremors are usually unilateral and occur when the affected segment is at rest and disappears when the patient makes any voluntary movement. Although it is the most characteristic symptom of PD, generally, tremors are one of the less disabling motor symptoms [2,3,4,5]. On the other hand, bradykinesia is a symptom characterised by slowness of motion and is one of the most relevant clinical features in PD. Bradykinesia results in difficulties with planning, initiation and execution of movements as well as performing sequential and simultaneous tasks. According to Jankovic [1], early bradykinesia symptoms are the slow implementation of the activities of daily living, the increase of the reaction time and slowness of motion. These manifestations can lead to difficulties in any tasks that require fine motor control (e.g., buttoning clothes or using tools). Bradykinesia is one of the most recognisable symptoms by clinicians in PD as it may become apparent before any formal neurological examination. Other PD motor symptoms are stiffness, postural alteration and freezing of gait (FoG) [1,6,7,8]. 

These symptoms, however, can be treated by means of specific medication that aims to increase dopamine concentration. Among current available therapies, levodopa-based ones are the most used since levodopa is the precursor of dopamine. At the beginning of levodopa treatment, patients present a meaningful response and symptoms may completely disappear for hours. However, these treatments only temporally revert the symptoms, but they do not prevent disease’s progression [9]. Hence, as disease progresses, dyskinesias and motor fluctuations appear. Firstly, levodopa-induced dyskinesia refers to exaggerated and involuntary movements occurring generally after prolonged treatment with levodopa. Dyskinesia term is applied to any involuntary movement with choreic nature (chorea), as a repetitive “*dance*”, or that provokes dystonic postures (dystonia) that typically affect trunk, limbs, neck or head. The occurrence of choreic dyskinesias is closely related to the levodopa levels in plasma: the highest levodopa-induced dyskinesia occurs when antiparkinsonian effects of levodopa are maximum [10,11,12,13]. Secondly, motor fluctuations refer to the oscillations between ON and OFF periods that patients present after few years of medication. OFF periods are considered as those parts of the day in which patients manifest PD symptoms, with the exception of dyskinesia. On the contrary, ON periods refers to the remaining time in which patients regain movement control and the only appreciable movement alteration is dyskinesia. Medication intakes are commonly scheduled to keep a constant dopamine level in order to maximise ON time without dyskinesia and minimise OFF periods and their duration [14,15]. 

Time in OFF is currently the main parameter employed to assess pharmacological interventions and to evaluate the efficacy of different active principles. Therefore, obtaining precise information on the long-term evolution of these ON-OFF fluctuations and their short-term alternations, i.e., onset and duration, is essential to provide optimal therapy to PD patients and minimise time in OFF and dyskinesias [14]. Currently, the only available method to collect such information consists of self-reporting diaries [16]. With this method, patients annotate their motor state every waking hour during 2 or 3 days. These diaries have some important shortcomings that limit their validity and their application in clinical practice. First, they have a recall bias, and, second, they present a reduced compliance [16]. In consequence, a wearable device capable of collecting PD motor fluctuations in an objective and reliable way could help to overcome the limitations of self-reporting diaries and, in addition, would provide clinicians with a valuable tool to reduce OFF time and dyskinesia of their patients. A system with these characteristics would result an invaluable tool in PD diagnosis. Early detection of dyskinesias and motor fluctuations would help to, first, enhance the effectiveness of the medication through a better regimen adjustment; second, to significantly improve the quality of life of patients and, third, to obtain a deeper understanding of the evolution of disease. Another area that could benefit from a tool with these characteristics is the clinical and epidemiological research. These studies are expensive and laborious and, often, these economic limitations affect the methodological rigor. Studies based on movement disorders are especially complicated, on the one hand by the lack of markers to establish a clear diagnosis and, on the other hand, by the lack of uniformity in diagnostic criteria. 

Wearable inertial sensors based on Micro-Electro-Mechanical Systems (MEMS) are the current technological basis to analyse PD symptoms. In this sense, Zwartjes et al. [17] analysed the automatic detection and severity assessment of tremor and bradykinesia in six PD patients. In this study, the following locations were used to place their wearable sensors: sternum, foot, thigh, and wrist. The methodology applied provided a good correlation with Unified Parkinson’s Disease Rating Scale (UPDRS) values. Similarly, Salarian et al. [18] employed two triaxial gyroscopes located on each of the forearms to detect and quantify tremor and bradykinesia in 20 PD patients, also obtaining a high correlation with related UPDRS values. Finally, some papers have been presented in the last years as a result of the work done under the European project PERFORM [19,20], where dyskinesia, tremor, and bradykinesia detection were analysed. So far, several limitations are found in former studies. Firstly, most of the experiments on assessing motor states took place in controlled settings (laboratory), where patients were asked to perform specific activities with the aim of eliciting PD symptoms [21,22,23,24]. These tests can barely provide information to the algorithm in order to confront false positives in real life and, hence, the algorithm loses consistency; Secondly, OFF states presented by PD patients are obtained, in most cases, by removing medication intakes prior to experiments. As a result, symptoms observed are easily more severe than what they would have in the daily life of patients, facilitating the discrimination between their presence or absence. Therefore, natural conditions to monitor fluctuations are preferred, avoiding any withdrawal of medication intake. 

This work presents a new method for the assessment of ON and OFF motor states through a single waist-worn device which has an accelerometer as primary sensor. The method estimates, on the one hand, the presence of bradykinesia based on a Support Vector Machine (SVM) classifier that detects gait followed by a specific signal processing method that identifies strides and, finally, a method for characterising bradykinesia. On the other hand, dyskinesia presence is determined by analysing specific frequency features of the inertial signal. Based on their outputs, ON and OFF motor states are assessed by a hierarchical algorithm. This algorithm is applied to the signals collected during 1 to 3 days from 15 patients during their daily activities in natural conditions, i.e., without removing any medication intake. Accuracies above 90% in detecting ON and OFF motor states are obtained with the hierarchical algorithm and the signals collected from a single sensor. 

The paper is organised as follows: the next section is devoted to reviewing the related works on sensors and algorithms for monitoring PD motor states. Section 3 and Section 4 present the methods and the signal processing techniques employed, respectively. Then, Section 5 reports the obtained results. Finally, our conclusions are detailed in Section 6.

## 2. Related Work

Many previous studies have focused on the detection of PD motor symptoms. More concretely, these works mainly rely on the use of inertial sensors, although there are other less extended alternatives, as the use of sEMG [25,26,27,28] or another physiological measure [29]. The majority of these research studies have employed wearable sensors to study specific PD symptoms such as FOG [30,31,32], dyskinesia [33,34,35,36] and bradykinesia [18,19,22,37,38,39]; thus, the aim was detecting the presence or severity of a certain symptom. However, the main objective of our work is the detection of ON/OFF motor states of patients, which is mainly related to a dopaminergic deficit and is not related to the presence of a unique symptom.

Previous works conducted so far on the detection of ON/OFF motor states are based on characterising PD symptoms by means of inertial sensors. This idea was followed by the work conducted in 2004 by scientists at the Medical Center at Leiden University [24], in which hypokinesia, tremor, and bradykinesia were characterised with the aim of determining the motor state in 50 PD patients wearing two wrist-worn accelerometers. In this study, three features were extracted in time periods of half an hour: time during which patients did not move, acceleration average and percentage of the time with tremor. Patient’s motor state was based on a decision tree; more specifically, it was determined by comparing the output of each feature against a threshold value. Through the usage of this method with the wrist-worn signals, the specificity and sensitivity achieved in detecting OFF states were of approximately 70%, being sensitivity referred to the accuracy in detecting OFF annotations and specificity to the ON ones.

Salarian et al. [40] conducted another study in which nine sensors were used by 13 patients during 3 to 6 h. These sensors, which consisted of seven gyroscopes and two accelerometers, were located at the trunk, shins, and forearms. Features extracted aimed to characterise different symptoms and movements: tremor, bradykinesia, as well as posture and gait parameters. The sensitivity achieved by using these features into time periods of 10 min and a logistic regression classifier was 76%, being the specificity 90%.

Some papers have been presented in the last years as a result of the work done under the European project PERFORM [19,20]. Cancela et al. collected data from 20 patients, while they developed a variety of activities. In these research works, a variety of machine-learning classifiers were analysed, obtaining classification errors of 14%. In addition, in another set of signals obtained from 24 PD patients at home during 7 days, the same method was applied, obtaining an error rate of 25.6% ± 14.9%. As part of the PERFORM project as well, Pastorino et al. [41] presented a preliminary result of the PERFORM project where a correlation between the algorithms developed in the project and motor states in two patients were reported. In this paper, the algorithms used were not specified and also the resulting specificity and sensitivity values are not available, only data correlation between the system output and patients’ diary were offered (88.2% ± 3.7%). In the most recent papers of the PERFORM project [42], this system was reported to present the ON/OFF related information based on UPDRS values. Nevertheless, in all cases, the system was composed of a set of 5 wearable sensors and a central storage unit, making the system, from our point of view, unusable as a continuous monitoring system in daily life. Table 1 summarizes the main works focused on the detection of ON-OFF motor states. 

Besides motor states, levodopa-induced dyskinesias, which are a side effect of the dopaminergic treatment, have been widely analysed. Many works have studied their automatic assessment based on MEMS sensors. Recently, Keijsers et al. employed five tri-axial acceleration sensors in 13 patients to achieve an accuracy of 96.6% in detecting dyskinesia [43]. The classification algorithm employed was based on artificial neural networks and labelled 15-min segments. In contrast, similar results were achieved by Tsipouras et al. but on smaller segments [44]. In this case, signals from four PD patients and six control subjects doing a number of previously scripted activities were collected. Patients wore eight sensors and several classifiers were evaluated on the signals obtained. The accuracies obtained ranged from 53.85% to 93.7%. 

Besides characterising motor symptoms, there is another approach that many research works have followed to evaluate the motor state of PD patients. This method is inspired in the assessment commonly done by neurologists at the clinical setting, which consists of administering a scale to patients that rates patients’ movements, e.g., UPDRS scale. In this approach, that takes advantage of the relationship between UPDRS and motor state [45], the goal is to assess PD patients while they are asked to do specific movements and they wear an inertial sensor or use a specific device. An example of such method is the Kinesia device, developed by Great Lakes Technology, which consists of a triaxial accelerometer and a gyroscope that measure patient’s finger movement providing values correlated with UPDRS [46]. This approach was also followed by the work of Patel et al. [22], who used eight sensors to estimate different UPDRS scores. Compared to the methods presented before, this one presents the shortcoming of requiring the patient to move in a specific way, as a finger tapping movement, necessary to provide the rating value. In consequence, the monitoring is discontinuous, requiring patients to stop their normal activity and may not be frequent enough to capture motor fluctuations. 

To sum up, some systems have been previously developed to monitor ON and OFF fluctuations during patient’s daily life; however, they require several IMU’s. Furthermore, almost all tests have been performed in laboratory conditions and, as previously mentioned, most works analyse OFF periods that were artificially induced through a prolonged withdrawal of the patients’ habitual medication. This commonly provokes deeper OFF periods than the natural ones and, hence, could result in non-real condition evaluation. In the next section, a new method for the assessment of motor states in PD patients based on a single waist-worn sensor is presented. This method is evaluated in 15 PD patients without medication withdrawal.

## 3. Methods

This section describes the methods used to validate the device and algorithms for the assessment of motor fluctuations. The section is organised as follows. First, the inclusion criteria and descriptive data of the patients who participated are described. In the data collection part, the data acquisition methodology is explained. 

### 3.1. Participants 

In this study, developed within the MoMoPa 2 project, a total of 15 patients with idiopathic PD, according to UK PD Society Brain Bank criteria [47], have been used to validate the algorithms. We included patients aged between 49 and 82 years old and patients who were in a mild or moderate stage of the disease (Hoehn and Yahr stage greater than or equal to 2.5 in their ON state) and had motor fluctuations with bradykinesia, freezing of gait or dyskinesia. Patients with other health problems that hamper physical activity and patients with dementia (DSM-IV-TR criteria) or neuropsychiatric disorders were excluded. The study was conducted in the province of Barcelona (Spain) over 3 years (from 2013 until 2015), the experimental protocol was approved by the local Ethics Review Committee and all participants provided informed consent prior to their inclusion in the study.

### 3.2. Sensor Device

**Figure 1:**
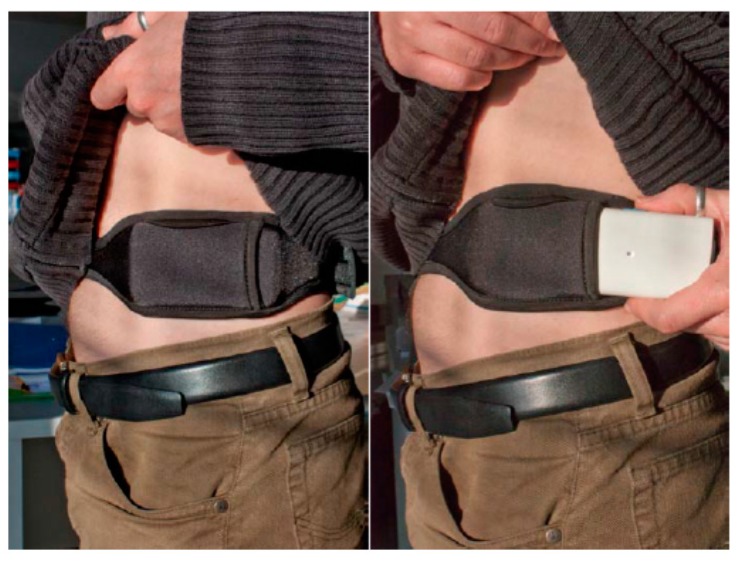
Image of the 9 × 2 sensor and the neoprene belt.

The device employed to collect inertial signals is the 9 × 2 device, which has been previously used to collect data from PD patients [48]. The device has been specifically designed for long-term monitoring and allows capturing inertial data as well as embedding algorithms in real-time. Thus, commercial devices are not suitable for the purpose of this work. This device saves the inertial measurements into a micro-SD card and includes a tri-axial accelerometer with a full-scale range of ±6 g. It also features a rechargeable Li-ion battery. The 9 × 2 maximum sampling data rate is 200 samples per second. The sensor is worn on the waist by means of a neoprene belt specifically designed for this purpose, as shown in Figure 1. 

Waist position has been selected due to the following rationale. Regarding the design and generation of a system and an algorithm for monitoring PD motor states, it is a key factor to determine the context and conditions in which the system will be used. According to this directive, two requirements lead and constrained the development of the algorithm and, consequently, the data acquisition required to generate the algorithms: the number and location of inertial sensors. We find, then, a trade-off between the feasibility of capturing accurate data, which affects the precision in recognising a motor symptom, and patient’s discomfort while wearing a sensor on certain positions or in simply having to wear several sensors. Thus, in this work, any system implementation that requires multiple sensors has been discarded and the study has been focused on the use of a single sensor. Another important issue is the position of the sensor and, in this sense, some studies like those of Yang et al. and Gjoreski et al. [49,50] have shown that placing an accelerometer on the waist provides good ergonomics for the patient. Furthermore, from a technical point of view, the closeness and solidarity with the centre of human body mass provide a precise characterisation of body movement. Along the same line, Mathie et al. [51] performed a study where volunteers chose, as the most suitable place to carry a small inertial sensor, the anterior superior iliac spine in the waist. Given this reasoning, in our work it was considered that a single sensor must be used and, in addition, it must be placed on the side of the waist, as depicted in Figure 1. Almost all human movements are reflected in the trunk and, among the different trunk locations a sensor could be located, the waist is considered to be the optimal place. Related to the symptoms that are detected by the sensor, as Section 4 presents, this location presents the advantage of enabling the detection of both bradykinetic gait and dyskinesia.

### 3.3. Data Collection 

Data collection is divided into two parts. The main database of accelerometer signals gathered from 15 PD patients was used to evaluate the motor state detection algorithms. These signals were collected with the device while patients freely performed their activities of daily life. Additionally, a former database obtained from 20 patients had been previously collected mainly in laboratory settings [52]. This database has been employed to develop the window-based analysis of the algorithms, while the first one has been used to validate the method. Both databases are presented in the following subsections.

#### 3.3.1. Evaluation Database of Inertial Signals

Data collection basically consists of three days of free monitoring, during which patients wore the 9 × 2 wearable device. The first day, early in the morning and prior to any recording, PD motor state was verified by clinical experts, by means of the motor section of the UPDRS, which was evaluated in order to objectively assess, with standard instruments, the characteristics of patients’ motor state. Then, a sensor was given to patients and its usage and location were described. Then, the researcher left patients’ home. This way, during the remaining of the first day, patients wore the sensor and did their usual daily life activities. In the morning of the second and third day, patients wore up the sensor by themselves and, as in the first day, performed their daily life activities in a regular way. In addition, patients used a diary over these three days to report their motor state every half an hour. 

Some specific cautions were taken to ensure the validity of the diaries reported by PD patients since they are known to present some shortcomings: first, patients may not correctly identify their motor state, and, second, non-motor symptoms could provoke wrong annotations [16]. In addition, time slots might be confused and time alterations in the diary could be introduced. The first caution taken was to check that patients enrolled correctly identified their motor state, which was done by doing some questions; The second measurement taken, which is the main one, consists in gathering an alternative diary. In this sense, a researcher called patients by phone every two hours while they used the sensor. This way, another register of patients’ motor state was obtained, which was used to verify the one delivered by patients. The third caution taken relies on assuming that the temporal validity of each annotation covers 15 min before and after the time of the annotation. Unfortunately, six patients stopped the experiment before the third day due to the inconvenience of filling the diary. The demographics, UPDRS score, Hoehn and Yahr stage and the dominant symptoms of the patients are provided in Table 2.

#### 3.3.2. Learning Database

The second set of accelerometer signals was gathered from 20 PD patients in laboratory setting as part of a study that took place during 2009 and 2010 [52]. Patients who participated were aged between 49 and 82 years of age, lived in the Barcelona (Spain) area and had been diagnosed to have idiopathic PD according to the criteria of the Brain Bank, London [47]. In this study, only patients with the mild or moderate stage of the disease and motor fluctuations were included.

Accelerometer signals were collected by a former version of the sensor device presented in Section 3.1. This device, that registered the signals in a micro-SD card, was worn by PD patients while they performed various activities in laboratory settings and outdoors. Laboratory activities comprised walking in a straight line, walking over an inclined plane, carrying a heavy object, setting a table and going upstairs and downstairs. The outdoors protocol consisted of a 15-min walk, at least. Patients who had motor fluctuations repeated the experiment, excluding the outdoors protocol, in OFF state, which was induced by avoiding the first-morning intake of medication. The experimental protocol was approved by the local Ethics Review Committee. 

## 4. Signal Processing Methods

The signal processing method applied to the waist-worn accelerometer measurements to determine the ON/OFF state of PD patients relies on characterising motor symptoms, similarly to the methods presented in the related work section. In this sense, two specific algorithms, which analyse the presence of dyskinesia and bradykinetic gait, are used. Their output is then merged based on a hierarchical algorithm that eventually provides the motor state estimation. This section describes each one of the methods used to estimate the motor state of PD patients. 

In a previous work of the authors [53], a previous version of the ON/OFF detector was presented, which was only based on the analysis patients’ gait and it was validated in a different database than the one used. This new work presents a novel approach in which two algorithms are combined: a bradykinetic gait detector and a choreic-dyskinesia detector that, combined by means of a decision tree, perform the detection of the motor states. Notably, the algorithmic basis for the detection of bradykinesia in [53] and this work, with some modifications, is based on a previous work by the authors [52]. On the other hand the algorithmic basis of the dyskinesia block is based on another previous work [33]. The way to combine these algorithms and the database where they are validated are completely new. In addition, the processing required for extending bradykinetic gait detection into 10-min periods and the method presented in Section 4.3 to self-tune the corresponding thresholds are new.

In our approach, ON/OFF states are estimated by means of dyskinesia and bradykinesia detection. The rationale of using these two symptoms is given below. First, motor fluctuations have been shown to be associated with oscillating levels of dopamine and to the appearance of PD motor symptoms [9]. In addition, bradykinesia has been identified as the motor symptom whose appearance is more closely related to the lack of dopamine [54]. More concretely, bradykinesia and OFF states are related to low dopamine levels. On the contrary, the motor alteration that correlates to high dopamine levels is peak-dose dyskinesias, which are linked to ON states [1]. In consequence, both bradykinesia and dyskinesia motor alterations are selected to determine ON/OFF motor states.

**Figure 2:**
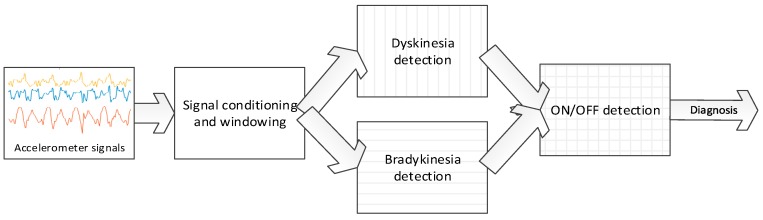
Block diagram of the dyskinesia, bradykinesia, and ON/OFF detection algorithms.

Taking into account these observations, we aim to obtain a set of signal processing methods that identify the presence of bradykinesia and dyskinesia during daily life activities, without requiring patients to perform specific movements, as some methods presented in the related work do. Related to choreic dyskinesia, this movement disorder can be evaluated without requiring any exercise, since it is an involuntary repetitive movement that patients manifest in any body segment with specific frequencies that have been shown to be up to 4 Hz [36,55]. Bradykinesia requires a more complex solution since, in general, it is manifested as a slower than normal movement that may be presented in any body segment. In order to automatically evaluate bradykinesia, movements belonging to the activities of daily living are considered, since they can be automatically assessed through wearable sensors. In this sense, gait is one of the movements involved in many of these activities and, in addition, it is an automatic movement that is also performed slowly by PD patients due to the effects of the disease [56]; hence, authors consider it the optimal way to analyse bradykinesia. This way, a signal processing method that analyses gait to determine the presence of bradykinesia is considered, which will enable the monitoring of low dopamine levels without requiring patients to do any specific movement. A general schematic representation of the algorithms is shown in Figure 2.

The next sections present the algorithms comprising the overall motor state detection: first, bradykinesia detection; second, dyskinesia detection and finally, the hierarchical algorithm that provides the motor state estimation based on the first two algorithms. Furthermore, to facilitate the understanding of the equations presented in the following sections, two tables are presented with the nomenclature of all the parameters (Table 3) and all the variables (Table 4) involved in the algorithms. Table 3 presents those parameters that are required to be tuned in order to properly detect the PD symptoms, while Table 4 summarises the variables that correspond to values that are computed by the algorithm from the inertial signals.

### 4.1. Dyskinesia Detection 

Dyskinesia detection is the first algorithm presented for the ON-OFF motor states detection. This algorithm was designed based on many previously works that relate dyskinesia to an increased power spectra of some specific frequency bands. The algorithm was developed by analysing the frequency spectra of inertial signals obtained from PD patients while performing different activities, either while presenting dyskinesias and without presenting them. As a result [52], a specific frequency band in which power spectra increases with dyskinesia was identified; in addition, other activities were found to also increase the power spectra in some bands that are overlapped with the dyskinesia one; thus, these other activities could provoke false positive detections of the symptom. These overlapping frequency bands are also examined in the algorithm in order to avoid inaccurate dyskinesia detections.

Dyskinesia frequency band was identified within the range of 0 to 4 Hz [57]. As mentioned previously, other activities with high power spectrum in the same band were found; for example, the natural frequency of gait and going upstairs and downstairs ranges from 0.5 to 6 Hz [58,59], being overlapped with the dyskinetic band. However, these activities have strong harmonics whose frequency reaches 20 Hz, which is not overlapped with the dyskinetic band. On the other hand, posture transitions span the band from 0 (not included) to 0.68 Hz [60]. A symptom that could introduce harmonics at frequencies of interest is tremor; nonetheless, according to a consensus of the Movement Disorders Society [55] the frequency of Parkinsonian tremor goes from 4 Hz to 9 Hz. This way, the upper limit of the dyskinesia band is set to the lowest frequencies of tremor, and the possible increment in the power spectra, caused by the tremor, would incorporate in the non-dyskinetic band. In consequence, dyskinesia algorithm relies on the calculus of three power spectra values: first, the one corresponding to dyskinetic band (*P_d_*), considered to be in the (0.68, 4] Hz range; second, non-dyskinetic band (*P_walk_*), considered to cover [8, 20] Hz; and postural transition band (*P_PT_*), which is (0, 0.68] according to [57]. The power spectra in a given band is computed as the summation of the corresponding harmonic amplitudes among the three axis.

The sampling frequency has been determined by the maximum frequency of interest following the Nyquist-Shannon sampling theorem [61]. Given a sample rate *f’_s_*, the complete reconstruction of a continuous signal is guaranteed for a frequency band limit below *f’_s_*/2. In consequence, *f’_s_* is set to 40 Hz, since 20 Hz is our maximum harmonic of interest in the previous frequency bands and we want to minimise the resources used by the algorithms. On the other hand, the window length is set to 128 samples since it enables the evaluation of postural transitions (below 0.68 Hz) and dyskinesias. In consequence, w = 128/*f*’_s_ = 3.2 s. 

Dyskinesia features *P_d_*, *P_walk_* and *P_PT_* are obtained in each window of 128 samples, i.e., 3.2 s. They are used, as Equation (1) shows, to determine if a patient manifests dyskinesia, does not manifest it, or performed a movement that does not allow to evaluate its presence (i.e., the output is *Unknown*). The latter case refers to a patient who walks or performs a postural transition, in which cases these movements do not enable the detection of dyskinesia since they have overlapped frequency bands. This way, the detection of dyskinesia in a certain window h is defined by $d_{h}^{w}$ according to:

(1) $$d_{h}^{w}=\{\begin{array}{cc} 1\;\left(Dyskinesia\right) & P_{d}>t_{d}\;\wedge \;P_{PT}<t_{PT}\;\wedge \;P_{walk}<t_{walk}\\ 0\;\left(No\;dyskinesia\right) & P_{d}\leq t_{d}\;\wedge \;P_{PT}<t_{PT}\;\wedge \;P_{walk}<t_{walk}\\ U\;\left(Unknown\right) & P_{PT}\geq t_{PT}\;\vee \;P_{walk}\geq t_{walk} \end{array}$$

where $t_{d}$, $t_{PT}$, $t_{walk}$ are the thresholds for dyskinetic and non-dyskinetic bands (posture transition band and walking band) respectively, ∨ is the logical *OR* operation and ∧ is the logical *AND*.

The values found for these thresholds are $t_{d}$ = 1.75, $t_{PT}$ = 0.95, and $t_{walk}$ = 1. These thresholds have been set based on the previously described study [52] (see Section 3.3.2) and they are used in a generic way for any patient.

Dyskinesia is a movement alteration that commonly appears during several minutes. Nonetheless, short windows are being used (w = 3.2 s). In order to determine the presence of dyskinesia in a more appropriate time interval, it is proposed to collect the output of several windows under a period of *T* = 60 s. Each window is overlapped with the previous one by 64 samples, i.e., a new window starts every half a window. The algorithm, thus, provides an output once per minute obtained from the information included in its ⌊2T/w⌋ windows, being ⌊·⌋ the floor function, according to:

(2) $$d_{j}^{m}=\{\begin{array}{cc} 1\;\left(Dyskinesia\right) & \overset{\left\lfloor 2T/w\right\rfloor }{\underset{h=1}{\sum }}\frac{d_{h}^{w}}{n_{d}}>t_{p}\;\wedge \;\frac{w\cdot n_{d}}{2T}>t_{c}\\ 0\;\left(No\;dyskinesia\right) & \overset{\left\lfloor 2T/w\right\rfloor }{\underset{h=1}{\sum }}\frac{d_{h}^{w}}{n_{d}}\leq t_{p}\;\wedge \;\frac{w\cdot n_{d}}{2T}>t_{c}\\ U\;\left(Unknown\right) & \frac{w\cdot n_{d}}{2T}\leq t_{c} \end{array}$$

where $d_{1}^{w},\ldots ,d_{\left\lfloor 2T/w\right\rfloor }^{w}$ are the outputs represented in Equation (1) corresponding to the windows evaluated in minute i, $n_{d}$ is the number of time windows in which the condition $P_{PT}\geq d_{PT}\;\vee \;P_{walk}\geq t_{walk}$ was not held, $t_{p}$ is the minimum rate of dyskinetic windows in the analysed period, and *t_c_* is the threshold that represents the minimum rate of windows to analyse in order to validate the detection.

According to Equation (2), the algorithm output in a one-minute interval is determined to be dyskinetic ($d_{j}^{m}=1$) provided that most of the analysed window outputs are dyskinetic. This means that a minute period is considered dyskinetic if the rate of positive outputs of Equation (1) in this period is greater than $t_{p}$. However, as these band’s power spectra might be increased by other activities, and not only by the appearance of dyskinesias, we must add a parameter of confidence in which we ensure that the patient is not performing activities that might cause false detections in this band. Then, a confidence index is defined as $\frac{w\cdot n_{d}}{2T}$ and represents the number of windows that were rejected due to the condition $P_{PT}<d_{PT}\;\vee \;P_{walk}<t_{walk}$. A low confidence index indicates an unreliable detection assessment because only few windows could be analysed. For this reason, the confidence index is required to be greater than threshold *t_c_*.

**Figure 3:**
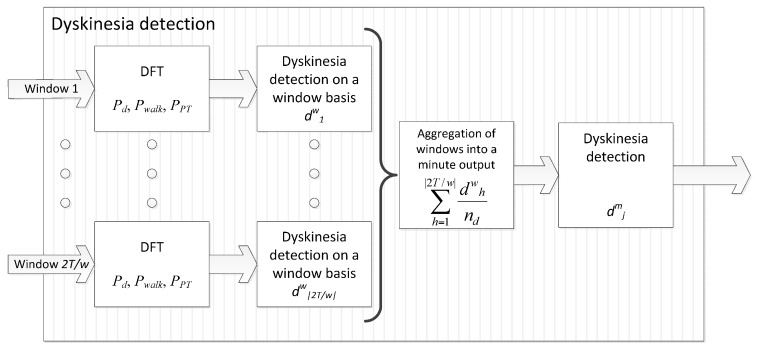
Block diagram of the dyskinesia algorithm.

Threshold values were found based on an optimisation procedure on the signals collected in the previously mentioned study with 20 patients [52], in which several values for the thresholds were evaluated in 10 patients and the accuracy was measured onto the signals from other 10 patients. Values found were $t_{p}$ = 0.4 and $t_{c}$ = 0.3. Figure 3 shows a schematic representation of the dyskinesia algorithm block.

### 4.2. Bradykinesia Detection 

Bradykinesia detection analyses Parkinsonian gait, as previously presented. The signal processing algorithm exploits the fact that gait, as an automated movement, is slowed in Parkinson’s patients during low-dopamine level periods. In consequence, it is considered that the signal processing method has to, first, determine that patients are walking; second, identify gait cycles from the accelerometer signals; and, third, characterise gait cycles through a measurement that correlates to the presence of bradykinesia. The complete bradykinesia detection method consists of a five-step characterisation method, as described below.

The first step consists in detecting gait and it is based on an SVM classifier. SVM is chosen, first, given the bi-classification nature of the problem at hand (detecting if the patient walks or not), which matches the bi-classification problems that SVM solve; second, due to the high performance that SVM have reported when dealing with this kind of classification problems; and, finally, because SVM allows us to obtain a global optimal solution, as opposed to Artificial Neural Networks that may provide suboptimal ones. Thus, given the signal contained in a time window of w = 3.2 s, as in the dyskinesia case, it is needed to determine whether the patient is walking or not. The SVM is trained with a Radial Basis Function (RBF) kernel and vectors {***p***_1_, …, ***p**_n_*}, where ***p**_i_* = [$h_{1}^{i},\ldots ,h_{k}^{i}$] and $h_{j}^{i}$ are the features which characterise the window of the accelerometer signal. Hyper-parameters γ and *C* were tuned through Cross-Validation by testing the following values: {10^−3^, 10^−2^, …, 10^3^}. In a previous study [52], 800 frequency features were analysed using data from 10 patients. Two features were finally selected for walking detection as those that maximised inter-class distance and minimised intra-class distance according to Relief algorithm [62]. These two features (*k* = 2) selected for gait detection were the tri-axial power spectra between in the frequency bands [0.1, 3] Hz and [0.1, 10] Hz, which are noted as *h_1_* and *h_2_*, respectively. 

Each vector ***p**_i_* has a label *y_i_* = {1, −1} according to video observations used to label the video: *y_i_* = 1 corresponds to those windows whose corresponding video labels were walking and *y*_i_ = −1 to the remaining windows. Dataset elements are denoted as {(***p***_1_, *y*_1_), …, (***p**_n_*, *y_n_*)} and were employed to find the SVM classifier that allows the detection of gait by solving:

(3) $$\begin{array}{c} \begin{array}{c} min_{z,b,\xi }\frac{1}{2}\;{\left\|z\right\|}_{2}^{2}+C\overset{N}{\underset{i=1}{\sum }}\xi_{i}\\ s.t\;\xi_{i}\geq 0 \end{array}\\ y_{i}\left[K\left(z,p_{i}\right)+b\right]\geq 1-\xi_{i} \end{array}$$

where $K\left(z,p_{i}\right)=e^{\gamma {\left\|z-p_{i}\right\|}_{2}^{2}}$, *b* is the hyperplane bias, z is the hyperplane that separates both classes and $\xi_{i}$ are the slack variables. Parameters *C* and γ are determined as the values that maximise the accuracy among the values 10^−2^, 10^−1^, …, 10^2^ in a 10-fold Cross-Validation [63], which were found to be 10 and 0.1, respectively. 

The label of a new window ***p*** is then obtained by:

(4) $$l\left(p\right)=sgn\;\left(\overset{l}{\underset{i=1}{\sum }}y_{i}\alpha_{i}K\left(p_{i},p\right)+b\right)$$

where the set of α are the Lagrangian multipliers of the dual problem formulation of the SVM.

The second phase is focused on detecting patients’ strides and only those windows whose feature representation ***p**_j_* = [$h_{1}^{j},h_{2}^{j}$] satisfies *l(**p**_j_)* = 1 (walking) are analysed. The principles used to detect strides are based on Zijlstra et al. work [64]. Although segmentation techniques can be employed to detect strides [65,66,67], we restrict to biomechanical properties of gait and the way they are observed in the acceleration signals to do so. More concretely, the beginning of the support phase of gait, that is when the heel touches the ground, can be detected by a local minimum in the front acceleration measured from the bottom of the trunk [64]. This event in the gait cycle is known as ‘initial contact’ and it is regarded as the beginning of step. However, due to lateral particularities of PD [1], our interest focuses on strides; i.e., the signal comprised between two consecutive steps of one feet. The discrimination between left and right steps can be performed by analysing the relative extrema of the lateral acceleration in the waist, which approximately describes a sinusoidal period during gait cycle [64]. 

The third step consists in characterising the detected strides in the previous phase with the aim of analysing the presence of bradykinesia. The basis of this step is the previously mentioned study [52] where several statistics were applied and evaluated in 20 patients. In this study, several features that characterised strides were analysed into their ability to linearly separate the presence of the interest symptom and, also, intuitively represent the fluidity of movement. From the conclusions of this work, the best feature to characterise the fluidity of movement was the power spectra in the bands of (0, 10] Hz of the stride. Given a stride detected on the accelerometer signal of a certain patient, the (0, 10] Hz power spectra of the stride is represented by $p_{j}^{str}$.

The considerations for the fourth step are related to the fact that bradykinesia is a symptom examined during gait, as it is an automatic movement. In consequence, with the aim of analysing gait during its highest degree of automation, the inertial parameters of gait are analysed after walking started and before the patient stopped. Therefore, we consider a sequence of *U* consecutive walking windows, i.e., *l* (**p**_*x*_) = −1, *l* (**p**_*x*+1_) = 1, …, *l* (**p**_*x*+*U*_) = 1, *l* (**p**_*x+U+1*_) = −1, called *walking stretch*, during which *S* strides are detected; first and last two strides are not considered because they are not done under a high automation control, i.e., only $S_{k}-$ 4 strides are considered. Thus, the result of averaging the fluency characteristics obtained from the strides within a walking stretch *k* is denoted by $f_{k}^{str}$ and is defined as:

(5) $$f_{k}^{str}=\frac{1}{S_{k}-4}\;\overset{S_{k}-2}{\underset{j=3}{\sum }}p_{j}^{str}$$

where $S_{k}$ is the number of strides detected in the walking stretch *k*. 

Considering that, when bradykinesia appears, it remains present for long periods of time, we designed an aggregation strategy that provides an algorithm output per minute, in order to simplify the final evaluation of the presence or absence of symptoms. In this aggregation, algorithm’s output for a given minute *h*, noted as $f_{h}^{m}$, is computed as the average of the bradykinesia values associated with the strides contained in the walking stretches within that minute. The standard deviation of these bradykinesia values is represented by $f_{h}^{s}$.

Finally, the algorithm’s fifth step aims to give more robust outputs every minute by considering longer periods. In consequence, a weighted average of the last 10 min is proposed. Note that the output of the algorithm persists once per minute. Weighted aggregation is based on the importance each bradykinesia value has; for example, the value obtained in a minute from only 2 strides should not have the same weight as the value obtained in one minute with 20 strides detected. Furthermore, a very high standard deviation within a minute means a large scatter in the data and, in case of very high values of $f_{h}^{s}$, the average may not be significant. Furthermore, it has been observed that when the patient goes upstairs or downstairs the standard deviation grows above the usual values. An estimation of the maximum value of $f_{h}^{s}$ has been empirically determined through the signals from the previous study [52]. This value was determined by studying the values of standard deviation presented by patients who had made the dubious activities. From the presented premises, values per minute are filtered in the following way: those minutes *h* in which $f_{h}^{s}$ is greater than threshold 1.7, which is formalised through $k_{h}$ coefficients that are set according to:

(6) $$k_{h}=\{\begin{array}{c} \begin{array}{cc} 1, & \left(f_{h}^{n}<2\right)\wedge (f_{h}^{s}<1.7)\\ 0, & otherwise \end{array} \end{array}$$

where $f_{h}^{n}$ is the number of steps in the minute *h.*

The results presented in [52] show that the algorithm works best when considering the average walking steps in stretches over 5 strides. From this result the value of the minimum number of steps in a minute to be able to estimate the presence of bradykinesia is 2. In order to take into account the number of strides, a weight function is included. The function selected is the sigmoidal, considering that the minimum weight (0) should be with 0 steps and the maximum weight is 1. Whereas the maximum number of strides in a minute will be around 30–40, it is considered that from 20 strides the confidence in this minute should be high and therefore the weight should be the maximum. According to these considerations, the weight of data $w_{h}$ in a minute *h* is represented by wh=11+e−fhn. Finally, the fluidity value representing a period of 1 min, but weighted with the last 10 min, is calculated through the next function:

(7) $${\hat{f}}_{j}^{m}=\frac{\sum_{h=j-9}^{j}\;f_{h}^{m}\;k_{h}w_{h}}{\sum_{h=j-9}^{j}k_{h}w_{h}}$$

where ${\hat{f}}_{j}^{m}=Unknown\;\left(U\right)$ if $\sum_{h=j-9}^{j}k_{h}w_{h}=0$.

This value is finally used to determine the bradykinesia presence in the last-minute period under analysis. Diagnosis of bradykinesia is set differently for the first minute. The existence of bradykinesia evaluated for the first minute ($b_{1}$) is defined by:

(8) $$b_{1}^{m}=\{\begin{array}{c} \begin{array}{cc} 1\;\left(Bradykinesia\right), & if\;{\hat{f}}_{1}^{m}<b_{th}^{m}\\ -1\;\left(No\;bradykinesia\right), & if\;{\hat{f}}_{1}^{m}\geq b_{th}^{m}\\ U\;\left(Unknown\right), & if\;{\hat{f}}_{1}^{m}=NaN \end{array} \end{array}$$

where $b_{th}^{m}$ is the patient-dependent threshold to determine the presence (1) or absence (−1) of bradykinesia. This threshold is unique for each patient and must be particularised, as it is described in next subsections.

From this first minute, in order to avoid constant changes of diagnosis in intermediate states, a minimum variation from the threshold must be considered. This minimum variation is consequently determined by the maximum allowable standard deviation. The presence or absence of bradykinesia for the next minutes (*j* > 1) is set as follows:

(9) $$b_{j}^{m}=\{\begin{array}{c} \begin{array}{cc} 1, & if\;{\hat{f}}_{j}^{m}<b_{th}^{m}-\frac{1.7}{2}\\ -1, & if\;{\hat{f}}_{j}^{m}>b_{th}^{m}+\frac{1.7}{2}\\ b_{j-1}^{m}, & if\;{b^{m}}_{th}+\frac{1.7}{2}\geq {\hat{f}}_{j}^{m}\geq b_{th}^{m}-\frac{1.7}{2}\\ U, & if\;{\hat{f}}_{j}^{m}=U \end{array} \end{array}$$

**Figure 4:**
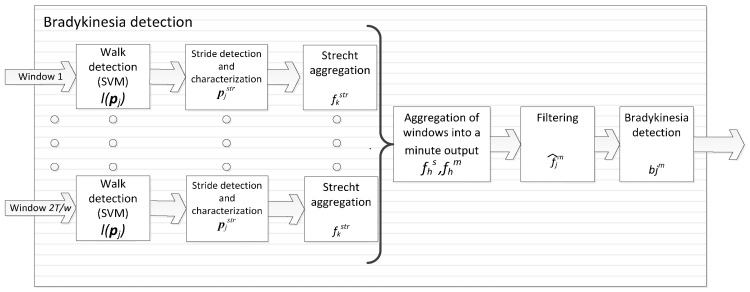
Block diagram of the bradykinesia algorithm.

Thus, the output of the bradykinesia algorithm in a given one-minute period *j*, noted as $b_{j}^{m}$, is U whenever the patient did not walk in the corresponding minute, $b_{j}^{m}=1$ in case of bradykinesia being detected, and $b_{j}^{m}=-1$ whenever not bradykinetic gait was present. Figure 4 shows a schematic representation of the bradykinesia algorithm block.

### 4.3. Self-Adapting Bradykinesia Detection Algorithm

The threshold applied to the output of the bradykinesia algorithm allows determining the presence of the symptom by dividing the range of possible values into two zones, one for each motor state. However, the selection of the threshold is very critical. Given that bradykinesia values $p_{j}^{str}$ depend on the way of walking of each individual, a young person without any pathological movement would provide high values; nonetheless, with older patients and/or with the presence of diseases such as arthritis, lower values would be obtained. Similarly, ON and OFF motor states are very patient-dependant. In consequence, bradykinesia values from each patient must be analysed in order to establish an optimal separation threshold.

In our previous works [15,52], the adaptation of the threshold to each patient was performed by a customisation process which is, in practice, long and complex. This process requires that the patient visits the clinical setting without medication, a fact which is already difficult to accomplish in many cases, but also implies that the medical team should perform a double clinical examination and a double assessment as the patient performs a series of exercises (mainly walking) both in OFF and ON states. Arguably, it is a methodology completely inapplicable in the clinical practice. In this section, a new methodology to calculate the threshold is presented.

This new methodology is based on automatically analysing the distribution of the bradykinesia fluency values (${\hat{f}}_{j}^{m}$) obtained during few days. Ideally, their histogram could present two clearly separate distributions representing each motor state. In this case, the bradykinesia optimal threshold lies within the gap between the two distributions. This type of clear distributions is found only in some patients, but it does not occur in most of them, where the difference between states is not so obvious. In these cases some empirical rules, that allow optimally adjusting the threshold, are applied.

More specifically, bradykinesia weighted values ${\hat{f}}_{j}^{m}$ are first collected during few days (from 1 to 3 days). A histogram is then obtained in order to analyse the data distribution. Histogram bins are arranged to cover bradykinesia values from 2 to 15 since, from our experience, fluidity values from PD patients in both motor states are contained within this range. Given this histogram, the special case in which two different distributions are found, i.e., one for each motor state, is determined by locating empty bins. In order to standardise it, it is considered that both distributions must be separated by at least 0.5 points and, in addition, both of them must contain at least 10% of the total data, in order to avoid identifying a double distribution from merely isolated data. For this case, the value of the threshold is set to that value in the middle between distributions.

However, the most common case consists of overlapping distributions. In this case, the premise for calculating the threshold for this group of patients consists in considering the lower values of the distribution corresponding to the OFF state and the highest ones to the ON state. Then, the threshold can be set based on the percentage of frequencies remaining on either side of the distribution. To implement this approach, the value of the histogram’s bin that has the largest absolute frequency, i.e., the mode, is obtained. Then, the bin that is located immediately below the mode and whose frequency is higher than 60% of the mode’s frequency, is selected as threshold $b_{th}^{m}$.

### 4.4. ON/OFF Motor States Detection

PD patients manifest motor fluctuations as an alternation between ON and OFF states. As previously described, provided that specific symptoms and movement alterations appear in each motor state, a hierarchical algorithm is designed to estimate the motor state of PD patients by combining the output of the previously presented methods. This way, dyskinesia and bradykinesia algorithms’ outputs are merged.

In order to work in a time unit closer to the gold standard, which are the annotations given by patients that are commonly provided every 30 or 60 min, a similar time basis is proposed. This time unit is 10 min since it is considered long enough to give accurate estimations and short enough to avoid mixing different motor states. More concretely, the motor state classifier first computes the presence of bradykinesia and dyskinesia into the period of 10 min, noted as $b_{i}^{10m}$ and $d_{i}^{10m}$, respectively, according to Equations (10) and (11):

(10) $$b_{i}^{10m}=\{\begin{array}{lll} U & \|b_{j}^{m}=U\|=10, & \forall \;j=i-9,\ldots ,i\\ -1 & \left(\left\|b_{j}^{m}=-1\|>\|b_{j}^{m}=U\right\|\right)\;\wedge & \\ & \|b_{j}^{m}=-1\|>2, & \forall \;j=i-9,\ldots ,i\\ 1 & (\left\|b_{j}^{m}=-1\right\|+\|b_{j}^{m}=U\|)<\|b_{j}^{m}=1\|\;\wedge & \\ & \|b_{j}^{m}=1\|>2, & \forall \;j=i-9,\ldots ,i\\ 0 & otherwise & \end{array}$$

(11) $$d_{i}^{10m}=\{\begin{array}{lll} U & \|d_{j}^{m}=U\|>7, & \forall \;j=i-9,\ldots ,i\\ 1 & \|d_{j}^{m}=1\|\geq 3, & \forall \;j=i-9,\ldots ,i\\ 0 & otherwise & \end{array}$$

where ‖·‖ counts the number of elements satisfying the within condition.

Bradykinesia-algorithm’s output $b_{i}^{10m}$ is 1 when the symptom is present, −1 if it is absent, 0 if an intermediate state has been detected and *Unknown* (*U*) if there is not any gait period detected in the last 10 min. On the other hand, dyskinesia-algorithm’s output $d_{i}^{10m}$ is 1 when the symptoms are present, 0 if absent and *Unknown* whenever the patient walked or performed a posture change in most of the 10 min, which is unlikely.

Once the 10-min output has been obtained, the motor state estimated by the algorithm at time $t_{i}$, which is noted as $v_{i}^{a}$, is defined by Equation (12):

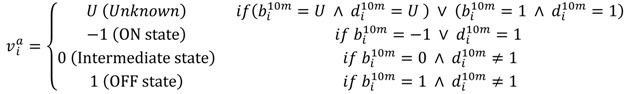
(12)


**Figure 5:**
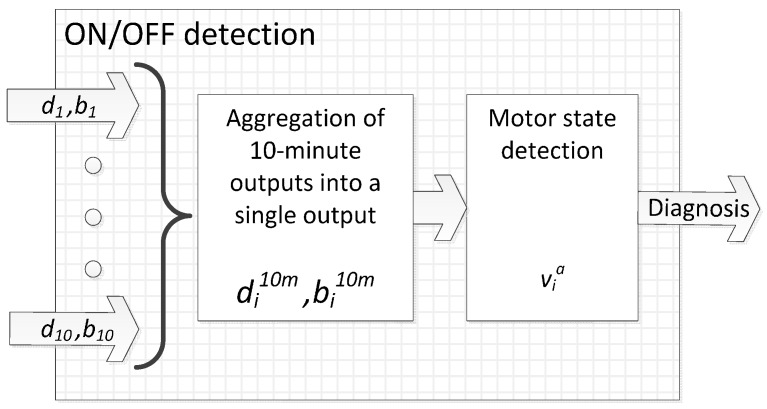
Block diagram of the ON-OFF algorithm.

OFF state is considered whenever a patient has a bradykinetic period. On the other hand, ON state is estimated if non-bradykinetic gait or dyskinesias have been detected. Finally, an intermediate state is also added as a consequence of the intermediate bradykinetic state. Once several consecutive outputs of the ON/OFF decision-tree defined in Equation (12) are obtained, a small filter is then applied. Considering three consecutive outputs of the decision-tree, if a blank period is found between two periods that are equal, the empty period is then set to the same state. Figure 5 shows a schematic representation of the complete ON-OFF algorithmic block.

### 4.5. Evaluation 

In order to formalise the analysis performed, the time of the patient annotation *i* corresponds to $t_{i}^{p}$ and it is defined by a value $v_{i}^{p}$ which corresponds to −1 for ON state, 0 for intermediate state (INT) and 1 for OFF state. The time corresponding to an output of the algorithm $v_{k}^{a}$ is noted as $t_{k}^{a}$. The meaning of $v_{k}^{a}$ is the same as $v_{k}^{p}$, corresponding the super-index *a* and *p* to the algorithm output and patient’s annotation, respectively. Since patients reported the motor state every 30 min, the validity period of an annotation of the patient is considered to be 15 min ahead and behind the time in which the patient wrote $t_{i}^{p}$. 

The output of the algorithm relies on the common statistical measures used in binary diagnostic tests. Thus, we consider a true positive (TP) when an OFF state is detected correctly and a false positive (FP) when an OFF state is diagnosed when the patient was in ON state. On the contrary, we consider a true negative (TN) when an ON state is detected correctly and false negative (FN) when an ON state is incorrectly obtained. It should be noted that intermediate states are excluded from the analysis since they cannot be identified as either of the motor states. Hence, the algorithm is evaluated based on the presented gold-standard; for each 10-min output of the algorithm $v_{i}^{a}$ that matches a diary annotation, a TP, FP, TN or FN is obtained according to:

(13) $$e_{i}=\left\{ \begin{array}{ll} TP & if\;v_{i}^{a}=1\;\wedge \;\exists \;k\;s.t.(v_{k}^{p}=1\;\wedge \;t_{i}^{p}-15\leq t_{k}^{a}-10\;\wedge \;t_{i}^{p}+15\geq t_{k}^{a})\\ FP & if\;v_{i}^{a}=1\;\wedge \;\exists \;k\;s.t.(v_{k}^{p}=-1\;\wedge \;t_{i}^{p}-15\leq t_{k}^{a}-10\;\wedge \;t_{i}^{p}+15\geq t_{k}^{a})\\ TN & if\;v_{i}^{a}=-1\;\wedge \;\exists \;k\;s.t.(v_{k}^{p}=-1\;\wedge \;t_{i}^{p}-15\leq t_{k}^{a}-10\;\wedge \;t_{i}^{p}+15\geq t_{k}^{a})\\ FN & if\;v_{i}^{a}=-1\;\wedge \;\exists \;k\;s.t.(v_{k}^{p}=1\;\wedge \;t_{i}^{p}-15\leq t_{k}^{a}-10\;\wedge \;t_{i}^{p}+15\geq t_{k}^{a}) \end{array}\right.$$

Finally, the sensitivity is the ability of an algorithm to correctly diagnose positive cases $Sensitivity=100\cdot \frac{\left\|\left\{e_{i}=TP\right\}\right\|}{\left\|\{e_{i}=TP\}\right\|+\left\|\{e_{i}=FN\}\right\|}$, while specificity is the ability of the algorithm to diagnose healthy cases $Specificity=100\cdot \frac{\left\|\{e_{i}=TN\}\right\|}{\left\|\{e_{i}=TN\}\right\|+\left\|\{e_{i}=FP\}\right\|}$. Specificity and sensitivity values are obtained for each patient.

## 5. Results and Discussion

A total of 420.2 h of inertial sensor signals were gathered from the 15 PD patients who participated in the experiment. Among these 15 patients, six decided to stop before concluding the three days of experimentation; however, some hours of valid data were obtained. More concretely, patients 1, 2 and 3 stopped the experiment after 24 h and the patients 4, 7 and 9 stopped after 48 h. All of them claimed that filling the diary, together with the calls, were very annoying. 

**Figure 6:**
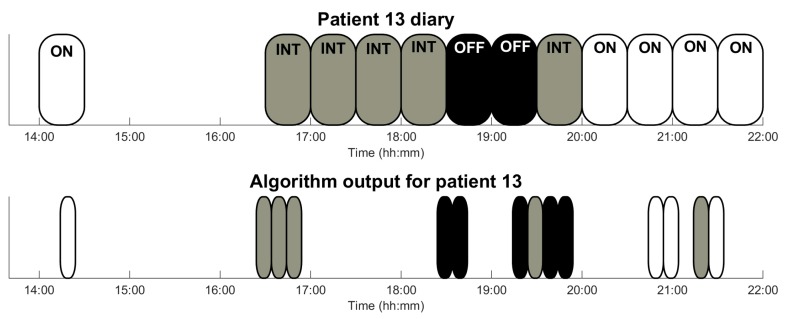

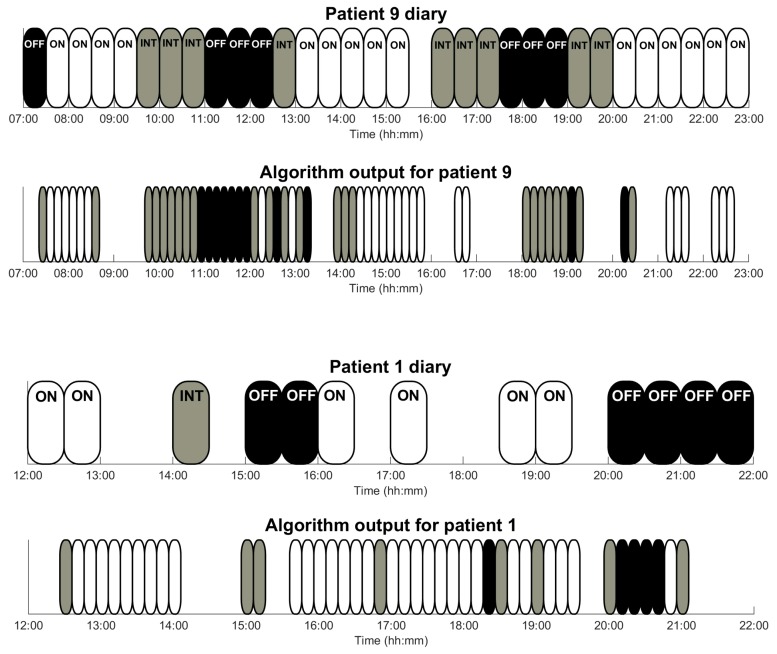
Graphical output of the algorithm for those patients who had the poorest results (patient 1) and some intermediate results (patients 9 and 13) during a day. Patient’s annotations (upper part) cover, each one of them, a time interval 30 min. Sensor outputs comprise 10 min and follow the same code colour: white corresponds to ON-state, grey to intermediate-state, and black to OFF-state.

As an example, Figure 6 presents, among others, the third day of patient 13: diary annotations reported by patients are depicted in the upper part and algorithm outputs, each one corresponding to a period of 10 min, in the lower part.

Table 5 shows, for each one of the 15 patients: accuracy, specificity and sensitivity of the OFF detection method; number of outputs provided by the ON/OFF method; number of outputs (Total outputs) that were used to compute specificity and sensitivity, since only those outputs with a diary annotation could be used; number of annotations reported by the patient (Total labels); the number of labels used in the validation (Labels used), since the algorithm output covers a period of 10 min and labels a time interval of 30 min; and the total number of minutes in the monitoring (Total of minutes). 

From Table 5, it is worth noting that the sensitivity average per-patient is 92% and the specificity is 92%. However, some patients (3, 5 and 14) do not present any OFF state detected by the algorithm, which is shown as NaN in the sensitivity column, since TP + FN = 0. These NaN values are obtained because these patients did not enter into the OFF state during the experiment or did not report it and, consequently, have no OFF state entries in the diaries. 

Patients reported an average of 26 annotations (13 h), meaning that the diary was far from being completely filled (approximately 60 annotations). However, patients 9 and 13 diaries are very complete in the number of entries (52 and 48 annotations respectively), which are very useful to validate the output of the algorithms in an optimal sense. In the case of patient 13, as can be seen in Figure 6, the output of the algorithm fully agrees with the annotations in the patient diary. Furthermore, in Table 5, some cases where a low number of daily patient labels have been validated are observed. For example, in the case of patient 15, two problems have been detected: on the one hand, the low number of entries (13 in 3 days) and, on the other hand, in OFF state, the patient could not or had no desire to move and, therefore, the algorithm was not able to provide a decision. In the case of patient 6, the problem is related to the high rate of intermediate states detected by the sensor. The algorithm output determines a great number of intermediate states because the patient walks briefly and also in very short stretches. This causes the algorithm not to be able to clearly evaluate gait and, thus, the motor state of the patient.

**Figure 7:**
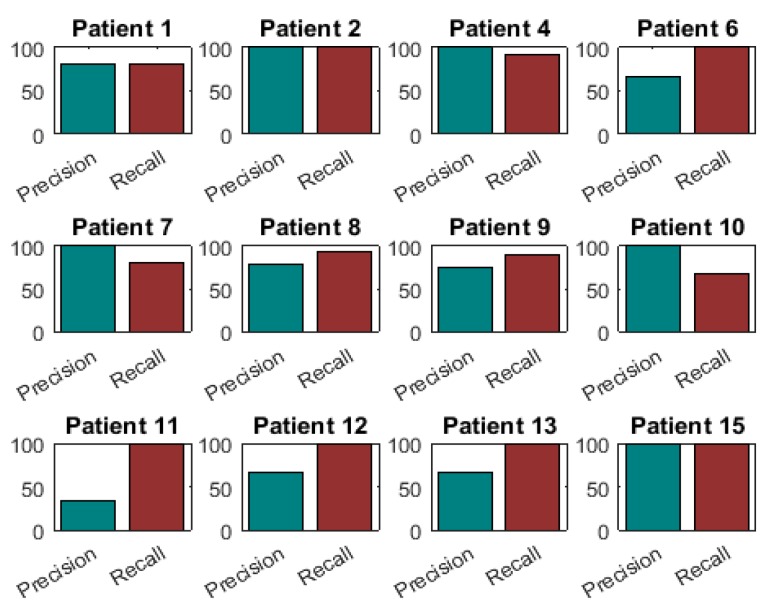
Precision-Recall diagrams of those patients who presented OFF states.

Figure 7 presents the precision-recall diagram of all patients who presented OFF state. This figure shows very high recall values in all patients, and somehow lower precision values for some of them. These low values (patients 11 and 6) are due to a low amount of OFF states, since only four and two OFF states are not detected (i.e., four and two false negatives), respectively. In patient 4 and patient 8, who have more than 10 OFF states, much higher values are obtained. 

The overall confusion matrix is presented in Table 6, considering the results among all patients. From this matrix, it follows that the method has an overall sensitivity and specificity of 90.28% and 92.11%, respectively, considering all the validated outputs of the algorithm, i.e., without distinguishing among patients. 

**Figure 8:**
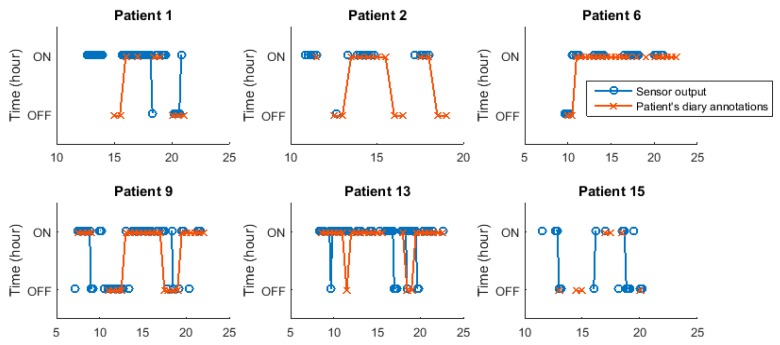
ON/OFF patterns for patients 1, 2, 6, 9, 13 and 15. Patterns obtained from the self-reported diaries and provided by the sensor are presented.

ON/OFF patterns have been previously used in the literature [39]. These patterns graphically summarize the ON/OFF pattern that patients have presented during one or more days. More specifically, they represent the most frequent motor state that patients annotated at each hour. Figure 8 presents the ON/OFF pattern for six patients who participated in the study. From this figure, it is observed that, with few specific exceptions, the estimations made by the algorithm mostly match the diary annotations.

So far, there are other works that also analysed the feasibility of monitoring PD motor fluctuations through wearable sensors. Nonetheless, previous works, as far as authors know, have used several sensors and, in most cases, in laboratory settings by following a set of scripted activities as shown in Table 1 [19,20,21,22,23]. The work of Cancela and Pastorino et al. developed in the project PERFORM [19] reports an accuracy of 86% [20] and 74.4% [19], respectively, with inertial signals obtained from twenty PD patients following a set of previously defined activities. These accuracies are similar or lower than the 92% reported in this work. Furthermore, their system is composed of a set of five wearable sensors and a central store unit, in contrast to the single device used in our study. Similarly, the work of Keijsers et al. and Patel et al. was performed with several inertial systems, being very cumbersome for patients [21]. Finally, it is difficult to compare the results obtained in this work with the one presented by Pastorino et al. in 2013, in which only two patients performed the test obtaining a 88.2% of correspondence between patient’s diary and ON/OFF phases identified by the system [41].

The main limitation of the algorithm presented in this work relies on the fact that the detection of OFF states can only be evaluated if patients walk. In consequence, there could be long periods during which the algorithm does not provide any information. In spite of this, it has been reported that PD patients in both moderate and advanced stages commonly walk more than 40 times per day [68,69]. Hence, it is considered that the algorithm would be capable of providing enough information. 

Currently, clinicians rely on patients’ self-reporting to monitor ON and OFF motor states. The algorithmic approach presented in this work combined with the simplicity of wearing a single waist-sensor has the potential of being an excellent clinical tool to replace these self-reporting diaries. However, the method requires further validation in more patients to confirm the results obtained in this research work.

## 6. Conclusions

In this work, a hierarchical algorithm has been presented, which combines the output of a dyskinesia-detection and a bradykinesia-detection method based on a waist-worn sensor in order to determine the motor state of PD patients. This algorithm has been validated in 15 PD patients with idiopathic Parkinson’s disease, who only wore a waist-sensor mainly consisting of a triaxial accelerometer. The results on sensitivity and specificity, above 90%, show the great potential of the method, both algorithm and sensor device, despite the rather low number of patients that has been validated. In addition, these results show that the motor status in PD patients is able to be monitored through a single sensor during daily life of patients in a precise and objective way. However, the method requires further validation in more PD patients.

This automatic detection of PD motor status might provide relevant advances. In a relatively short time, physicians can obtain accurate information for the purpose of adjusting the medication intake. Furthermore, this automatic assessment opens up the possibility to modify drug infusion rates in apomorphine and duodopa pumps in real-time, by adjusting to patients’ motor state. In addition, clinical trials may benefit from such tool since an objective comparison of the efficacy of distinct active principles would be obtained. 

To conclude, wearable devices for PD patients that objectively monitor the disease in daily life environments are a great advance in the clinical practice. Through them, the pharmacological regimen can be tailored to PD patients and, in addition, drug infusion pumps can interact in real-time to improve patients’ motor state. 

## Figures and Tables

**Table 1:** Summary of most relevant ON-OFF works.

Authors	Year	Number of Patients	Number of Sensors	Time Assessment	ON-OFF Results
Pastorino et al. [41]	2013	2	5	4 h, 2 days, unscripted activities	88.2% correspondence with UPDRS scales
Pastorino et al. [19]	2011	24	5	Scripted activities	74.4% accuracy
Cancela et al. [20]	2010	20	5	Specific movements	Brad. detection: 70% (walking), 86.6% (upper limbs)
Keijsers et al. [21]	2006	23	6	3 h activities, laboratory settings	96% sensitivity, 95% specificity
Patel et al. [22]	2009	12	8	Specific movements	Error: 3.4% in tremor, 2.2 in brad, and 3.2% in dysk
Hoff et al. [24]	2004	50	2	One hour and a half	70% accuracy

**Table 2:** Demographics and PD symptoms of the patients included in the database.

Patient	Age	H & Y	Gender	UPDRS/Motor State	Dyskinesia	Motor Fluctuations	Bradykinesia	Rigidity	Tremor	Postural Instability	FoG
1	61	2.5	Female	29/OFF	✓	✓	✓	✓	✓	✓	
2	59	3	Female	46/OFF	✓	✓	✓	✓		✓	✓
3	70	3	Female	29/OFF	✓	✓	✓	✓		✓	✓
4	49	2.5	Male	19/INT	✓	✓		✓	✓	✓	✓
5	68	2.5	Male	16/INT	✓	✓	✓	✓	✓	✓	✓
6	80	2.5	Male	11/ON		✓	✓	✓		✓	✓
7	63	2.5	Female	38/INT	✓	✓	✓	✓	✓	✓	✓
8	57	2.5	Male	6/ON		✓		✓		✓	✓
9	61	2.5	Male	25/OFF		✓	✓	✓	✓	✓	✓
10	66	2.5	Male	17/INT	✓	✓		✓	✓	✓	✓
11	64	4	Male	62/OFF	✓	✓	✓	✓	✓	✓	✓
12	63	2.5	Male	7/ON	✓	✓	✓			✓	✓
13	57	2.5	Male	9/ON		✓	✓	✓		✓	✓
14	60	2.5	Female	8/ON		✓	✓			✓	✓
15	59	2.5	Male	11/INT	✓	✓	✓	✓		✓	✓

**Table 3:** Summary of the parameters used in this work.

Parameter	Algorithm	Description	Value
$t_{d}$	Dyskinesia	Threshold for dyskinetic band	1.75
$t_{PT}$	Dyskinesia	Threshold for postural transition band	0.95
$t_{walk}$	Dyskinesia	Threshold for walk band	1
$t_{p}$	Dyskinesia	Threshold for the probability of dyskinesia occurrence in 1 min	0.4
$t_{c}$	Dyskinesia	Threshold for the confidence of dyskinesia occurrence in 1 min	0.3
C	Bradykinesia	Balance between empirical error and hyperplane margin	10
γ	Bradykinesia	RBF kernel hyper-parameter	0.1
α, z, b, $\xi_{i}$	Bradykinesia	SVM model. Obtained by solving the SVM-related optimization process	-
$b_{th}^{m}$	Bradykinesia	Patient-dependent fluency threshold to determine the presence or absence of bradykinesia.	Self-tuned (see Section 4.3)

**Table 4:** Summary of the variables used in this work.

Variable	Algorithm	Description
$P_{d}$	Dyskinesia	Power spectra in dyskinetic band
$P_{PT}$	Dyskinesia	Power spectra in postural transition band
$P_{walk}$	Dyskinesia	Power spectra in walk band
$d_{h}^{w}$	Dyskinesia	Dyskinesia detection in window *h*
$d_{j}^{m}$	Dyskinesia	Dyskinesia detection in the *j*-th 1-min period
$d_{i}^{10m}$	ON/OFF	Dyskinesia detection in the *i*-th 10-min period
$n_{d}$	Dyskinesia	number of time windows in which the condition $P_{PT}\geq d_{PT}\;\vee \;P_{walk}\geq t_{walk}$ was not held
$p_{i}$	Bradykinesia	Vector of the features that characterize the window of the accelerometer signal (for walking detection)
$y_{i}$	Bradykinesia	Window label according to video observations (for walking detection)
l(p)	Bradykinesia	SVM output (walk/no walk) for a given window represented by p
$p_{j}^{str}$	Bradykinesia	Power spectra of the stride *j*
$S_{k}$	Bradykinesia	Number of strides detected in the walking stretch *k*
$f_{k}^{str}$	Bradykinesia	Averaged fluency value for the strides within the walking stretch *k*
$f_{h}^{m}$	Bradykinesia	Averaged fluency value of the strides done within minute *h*
$f_{h}^{s}$	Bradykinesia	Standard deviation of the fluency values corresponding to the strides done in the minute *h*
$f_{h}^{n}$	Bradykinesia	Number of strides analyzed in minute *h*
${\hat{f}}_{j}^{m}$	Bradykinesia	Fluency weighted value for minute *j*
$k_{h}$	Bradykinesia	Filtering coefficient for minute *h*
$w_{h}$	Bradykinesia	Weight for fluency value in minute *j*
$b_{j}^{m}$	Bradykinesia	The existence of bradykinesia evaluated for minute *j*
$b_{i}^{10m}$	ON/OFF	Bradykinesia detection in the *i*-th 10-min period
$v_{k}^{a}$	ON/OFF	Motor state estimation done by the algorithm in the *k*-th 10-min period
$t_{k}^{a}$.	ON/OFF	Time of the *k*th motor state estimation done by the algorithm (corresponding to the first minute of the 10-min period)
$v_{i}^{p}$	ON/OFF	*i*-th motor state annotation given by a patient that corresponds to time $t_{i}^{p}$
$t_{i}^{p}$	ON/OFF	Time of the annotation *i* given by a patient

**Table 5:** Results organised by patient. The number of outputs given by the sensor and algorithm are presented in the ‘Total outputs’ column, distinguishing among ON, OFF and INT states. The number of Unknown outputs is shown as “Unknown”, being in brackets the amount corresponding to a detection of both bradykinesia and dyskinesia “(n. br. + dy.)”.

Patient	Accuracy	Specificity	Sensitivity	TP/TN/FP/FN	Total Outputs (ON/OFF/INT)	“Unknown” (n. br.+dy.)	Outputs Used	Total Labels	Labels Used
1	81.82%	83.33%	80.00%	4/5/1/1	19 (6/5/8)	7 (0)	11	10	7
2	100.00%	100.00%	100.00%	1/15/0/0	29 (15/1/13)	14 (0)	16	16	8
3	100.00%	100.00%	NaN	0/27/0/0	34 (27/0/7)	21 (0)	27	19	13
4	94.74%	100.00%	92.31%	12/6/0/1	38 (7/12/19)	21 (0)	19	22	10
5	91.89%	91.89%	NaN	0/68/6/0	102 (68/6/28)	25 (0)	74	44	33
6	87.50%	83.33%	100.00%	4/10/2/0	53 (10/6/37)	37 (0)	16	33	9
7	92.31%	100.00%	80.00%	4/8/0/1	19 (9/4/6)	7 (0)	13	9	7
8	83.87%	73.33%	93.75%	15/11/4/1	48 (12/19/17)	27 (0)	31	30	15
9	93.33%	94.00%	90.00%	9/47/3/1	93 (48/12/33)	51 (0)	60	52	33
10	83.33%	100.00%	66.67%	4/6/0/2	23 (8/4/11)	31 (0)	12	25	9
11	85.19%	84.00%	100.00%	2/21/4/0	34 (21/6/7)	20 (0)	27	24	14
12	92.59%	91.30%	100.00%	4/21/2/0	37 (21/6/10)	20 (2)	27	25	16
13	95.83%	95.45%	100.00%	2/21/1/0	42 (21/3/18)	94 (0)	24	48	17
14	91.67%	91.67%	NaN	0/11/1/0	19 (11/1/7)	30 (0)	12	21	10
15	100.00%	100.00%	100.00%	4/3/0/0	9 (3/4/2)	18 (1)	7	13	4

**Table 6:** Confusion matrix summarising the results from all patients.

		Predicted		
		Positive	Negative	
**Real**	**Positive**	65	7	72
	**Negative**	24	280	304
		89	287	
